# Outcomes of COVID-19 among Patients on In-Center Hemodialysis: An Experience from the Epicenter in South Korea

**DOI:** 10.3390/jcm9061688

**Published:** 2020-06-02

**Authors:** Hee-Yeon Jung, Jeong-Hoon Lim, Seok Hui Kang, Seong Gyu Kim, Yong-Hoon Lee, Jaehee Lee, Hyun-Ha Chang, Shin-Woo Kim, Ji-Young Choi, Jang-Hee Cho, Chan-Duck Kim, Yong-Lim Kim, Sun-Hee Park

**Affiliations:** 1 Division of Nephrology, Department of Internal Medicine, School of Medicine, Kyungpook National University, Kyungpook National University Hospital, Daegu 41944, Korea; hy-jung@knu.ac.kr (H.-Y.J.); jh-lim@knu.ac.kr (J.-H.L.); jyss1002@hanmail.net (J.-Y.C.); jh-cho@knu.ac.kr (J.-H.C.); drcdkim@knu.ac.kr (C.-D.K.); 2 Division of Nephrology, Department of Internal Medicine, Yeungnam University Medical Center, Daegu 42415, Korea; kangkang@ynu.ac.kr; 3 Division of Nephrology, Department of Internal Medicine, Daegu Catholic University Medical Center, Daegu 42472, Korea; shaorangun@gmail.com; 4 Division of Pulmonary and Critical Care Medicine, Department of Internal Medicine, School of Medicine, Kyungpook National University, Kyungpook National University Hospital, Daegu 41944, Korea; id0121@naver.com (Y.-H.L.); jaelee@knu.ac.kr (J.L.); 5 Division of Infectious Disease, Department of Internal Medicine, School of Medicine, Kyungpook National University, Kyungpook National University Hospital, Daegu 41944, Korea; changhha@knu.ac.kr (H.-H.C.); ksw2kms@knu.ac.kr (S.-W.K.)

**Keywords:** COVID-19, SARS-CoV-2, hemodialysis, South Korea

## Abstract

Patients with advanced chronic kidney disease (CKD) or who are on hemodialysis (HD) could have increased susceptibility to the 2019 coronavirus disease (COVID-19) given their pre-existing comorbidities, older age, compromised immune system, and regular visits to populated outpatient dialysis centers. This study included 14 consecutive patients on HD or with advanced CKD who initiated HD after being diagnosed with laboratory-confirmed COVID-19 from February to April 2020 in hospitals throughout Daegu, South Korea. The included patients, 42.9% of whom were men, had a mean age of 63.5 years. Four patients had a history of contact with a patient suffering from COVID-19. The most common symptom was cough (50.0%), followed by dyspnea (35.7%). The mean time from symptom onset to diagnosis and admission was 2.6 and 3.5 days, respectively. Patients exhibited lymphopenia and elevated inflammatory markers, including C-reactive protein and ferritin. Chest radiography findings showed pulmonary infiltration in 10 patients. All patients underwent regular HD in a negative pressure room and received antiviral agents. Four patients received mechanical ventilation and continuous renal replacement therapy at a median duration of 14.0 and 8.5 days, respectively. One patient underwent extracorporeal membrane oxygenation for three days. Among the 14 patients included, two died due to acute respiratory distress syndrome, nine were discharged from the hospital, and three remained hospitalized. Despite the high-risk conditions associated with worse outcomes, patients on HD did not exhibit extremely poor overall COVID-19 outcomes perhaps due to early diagnosis, prompt hospitalization, and antiviral therapy.

## 1. Introduction

An outbreak of the novel coronavirus disease 2019 (COVID-19), caused by severe acute respiratory syndrome coronavirus-2 (SARS-CoV-2) in Wuhan, China, has spread rapidly worldwide, including Daegu, South Korea, and has become an international health concern [1,2,3,4]. Reports have suggested that older age and comorbidities may be risk factors for poor outcomes [5,6]. Accordingly, chronic kidney disease (CKD) has been associated with a three-fold overall increase in the risk for severe COVID-19 [7]. In particular, patients with advanced CKD or who are on hemodialysis (HD) could have increased susceptibility to COVID-19 for the following reasons [8,9]: patients undergoing dialysis are generally older and have various comorbidities, such as diabetes mellitus, hypertension, and cardiovascular diseases, which have been known to negatively impact COVID-19 outcomes [5,10]. Patients with uremia and on dialysis are considered to be in a state of immune dysfunction with alterations in both innate and adaptive immunity, which may promote increased risk for infections. For patients who receive in-center HD and who regularly visit populated outpatient dialysis centers, social distancing and self-quarantine at home are impossible during the COVID-19 pandemic. As such, COVID-19 may have a considerably negative impact on both personal and social aspects of patients on dialysis. Early detection of symptoms related to COVID-19 among patients on dialysis, with prompt diagnosis and implementation of isolated dialysis, is crucial for reducing mortality and preventing disease transmission within the dialysis center and the community. To date, only a few reports regarding the clinical characteristics and outcomes of COVID-19 among patients on HD are available [11,12,13,14,15,16].

In this paper, we report the clinical characteristics and outcomes of COVID-19 in 14 patients on HD in Daegu, South Korea.

## 2. Methods

### 2.1. Patients

Consecutive patients on HD or with advanced CKD who were diagnosed with COVID-19 and transferred to Kyungpook National University Hospital, Daegu Catholic University Medical Center, Yeungnam University Medical Center and Daegu Fatima Hospital in Daegu in South Korea from February to April 2020 were included herein. Those receiving maintenance HD throughout all HD centers in Daegu and those with advanced CKD who initiated HD after being diagnosed with COVID-19 were included. The study protocol was reviewed and approved by the Institutional Review Board of Kyungpook National University Hospital (2020-04-059). Informed consent was waived due to the retrospective design of the current study. Patient information was anonymized prior to analyses.

### 2.2. Laboratory Confirmation of SARS-CoV-2

Nasopharyngeal and oropharyngeal swabs were obtained from the patients, after which real-time reverse transcriptase polymerase chain reaction was conducted to detect SARS-CoV-2. RNA was extracted from clinical samples using Allplex™ 2019-nCoV assay (Seegene, Seoul, Korea) according to the manufacturer’s instructions.

### 2.3. Data Collection

Baseline information collected upon admission included age, sex, dialysis duration, cause of end-stage renal disease, body mass index, comorbidities, history of contact with patients suffering from COVID-19, symptoms, laboratory data, vital signs, and chest radiography findings. Comorbid conditions included a history of diabetes mellitus, hypertension, congestive heart failure, coronary artery disease, cerebrovascular disease, chronic obstructive pulmonary disease, and malignancy. Laboratory data included C-reactive protein, ferritin, procalcitonin, hemoglobin, white blood cell count, lymphocyte count, platelet count, albumin, alanine aminotransferase, aspartate aminotransferase, creatine phosphokinase, lactate dehydrogenase, and lactic acid.

### 2.4. Criteria for Quarantine Release and Discharge

The criteria for quarantine release and discharge included the following: (1) clinical improvement of symptoms and (2) two laboratory-confirmed negative results for SARS-CoV-2 conducted at least 24 h apart.

### 2.5. Statistical Analysis

The Kolmogorov–Smirnov test was used to determine the normality of distribution for continuous variables. Continuous variables are presented as means ± standard deviations or median (interquartile range) as appropriate, whereas categorical variables are presented as a number (percentage). Imputation was not performed for missing data. All statistical analyses were performed using SPSS Statistics for Windows, Version 22 (IBM Corp., Armonk, NY, USA), with *p* < 0.05 being considered statistically significant.

## 3. Results

### 3.1. Clinical Characteristics

Table 1 details the clinical characteristics of the included patients. The mean age was 63.5 years with the oldest being 88 years old, and 42.9% of the patients were men. Mean dialysis duration was 4.7 ± 5.3 years. Among the included patients, 7 (50.0%), 11 (78.6%), 2 (14.2%), and 3 (21.4%) had diabetes mellitus, hypertension, cardiovascular disease, and cerebrovascular disease, respectively, while two (14.3%) and four (28.6%) patients had a history of cancer and a history of contact with a patient suffering from COVID-19, respectively. Patients had a mean Charlson Comorbidity Index of 5.6. The most common symptom was cough (50.0%), followed by dyspnea (35.7%), fatigue (28.6%), and sputum production (21.4%). The mean durations from symptom onset to diagnosis and admission were 2.6 and 3.5 days, respectively.

### 3.2. Clinical Findings

**Figure 1:**
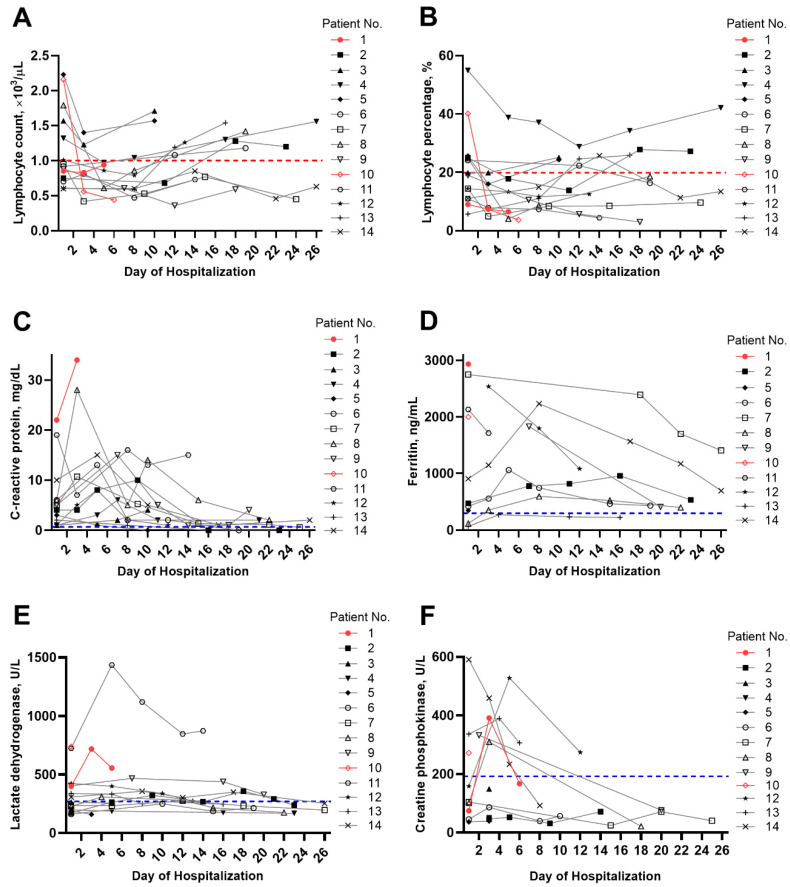
Temporal changes in laboratory markers in individual patients with end-stage renal disease and COVID-19. The temporal changes for (**A**) lymphocyte count, (**B**) lymphocyte percentage, (**C**) C-reactive protein, (**D**) ferritin, (**E**) lactate dehydrogenase, and (**F**) creatine phosphokinase. The red symbols and solid red lines indicate the results for patients who died. The dotted red lines indicate the lower limit of normal for lymphocyte count and lymphocyte percentage, while the dotted blue lines indicate the upper limit of normal for each parameter.

**Figure 2:**
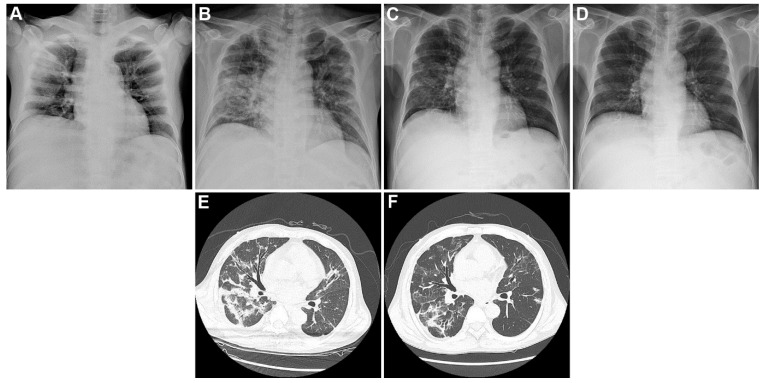
Temporal changes in chest imaging studies in one patient with end-stage renal disease and COVID-19. Temporal changes in chest radiography and computed tomography for patient number 2. Initial chest radiography upon admission showed focal consolidations in the right upper and left middle lung zones (**A**). (**B**,**E**) Images obtained on the 16th day of hospitalization showed bilateral worsening of multifocal patchy consolidations and ground glass opacities. (**C**,**F**) Images obtained on the 23rd day of hospitalization (the day of discharge) showed improvement of multifocal patchy consolidations and ground glass opacities, as well as organized changes. (**D**) Image obtained on 14th day after discharge (37 days from admission) showed resolved inflammatory lung lesions.

Table 2 summarizes the vital signs, laboratory data, and imaging findings upon admission. Among the included patients, two (14.3%), five (35.7%), and three (21.4%) had tachycardia, tachypnea, and fever upon hospitalization, respectively. Patients showed elevated median levels of C-reactive protein (6.1 mg/dL, range: 3.0–8.8 mg/dL), ferritin (472.0 ng/mL, range: 292.4–1693.2 ng/mL), procalcitonin (0.6 ng/mL, range: 0.3–1.0 ng/mL), and D-dimer (1.7 µg/mL, range: 1.0–3.8 µg/mL). Most patients exhibited lymphopenia. Figure 1 shows the temporal changes in laboratory markers, including (Figure 1A) lymphocyte count, (Figure 1B) lymphocyte percentage, (Figure 1C) C-reactive protein, (Figure 1D) ferritin, (Figure 1E) lactate dehydrogenase, and (Figure 1F) creatine phosphokinase. Accordingly, C-reactive protein, ferritin, lactate dehydrogenase, and creatine phosphokinase levels tended to decrease over time, whereas the lymphocyte count and lymphocyte percentage tended to increase over time. Chest radiography findings showed pulmonary infiltration in 10 patients. Representative chest radiography and computed tomography findings of two patients are presented in Figure 2.

### 3.3. Treatments and Outcomes

All patients received antiviral treatment, including lopinavir and ritonavir or hydroxychloroquine. Among the included patients, 7 (50.0%), 13 (92.9%), 2 (14.3%), 3 (21.4%), and 1 (7.1%) received hydroxychloroquine, antibiotics, antifungal treatment, glucocorticoids, and intravenous immunoglobulin, respectively (Table 3). All patients received regular HD in a negative pressure room. During HD, three (21.4%) patients experienced vascular access occlusion and received temporary vascular access, whereas four (28.6%) and two (14.3%) patients needed low-flow and high-flow oxygen therapy, respectively. Four patients (28.6%) received mechanical ventilation and continuous renal replacement therapy at a median duration of 14.0 and 8.5 days, respectively, whereas one patient (7.1%) received extracorporeal membrane oxygenation for 3 days (Table 3).

**Figure 3:**
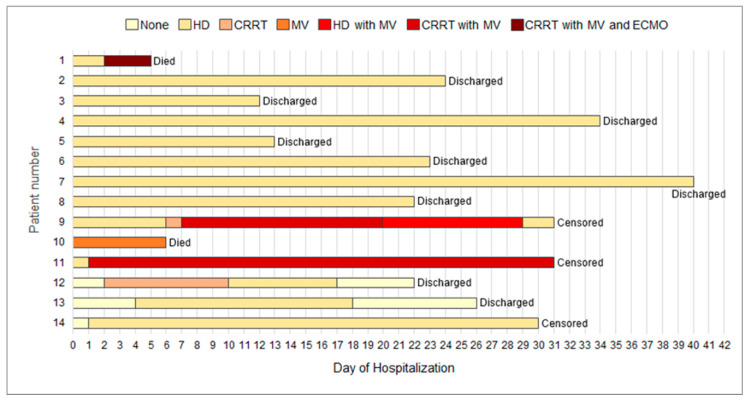
Clinical courses and outcomes for individual patients with end-stage renal disease and COVID-19. As of 14 April 2020, a total of 2 (14.3%) patients had died, 9 (64.3%) had been discharged, and 3 (21.4%) remained under treatment. Abbreviations: COVID-19, coronavirus disease 2019; HD, hemodialysis; CRRT, continuous renal replacement therapy; MV, mechanical ventilation; ECMO, extracorporeal membrane oxygenation.

Median lengths of hospital and intensive care unit stay were 22.0 and 6.0 days, respectively, whereas the median duration of positive COVID-19 test results was 21 days. Since the discovery of the first COVID-19 case in a patient on HD in Daegu, on 18 February 2020 until 14 April 2020, two (14.3%) patients died due to acute respiratory distress syndrome, nine (64.3%) were discharged from the hospital, and three (21.4%), including one in critical condition, remained hospitalized (Table 4). During the same period, 24 patients on HD without COVID-19 (4.1%) died out of 582 patients who were on maintenance HD at all centers. Clinical outcomes for individual patients are presented in Figure 3.

## 4. Discussion

The present study describes the clinical characteristics and outcomes of 14 patients on in-center HD who had been diagnosed with laboratory-confirmed COVID-19, which may be one of the largest studies involving such patients to date. Among the 14 cases included, 11 had received maintenance HD for a mean duration of 4.7 years and 3 patients with stage 5 CKD initiated HD after being diagnosed with COVID-19. Among the three incident HD cases, one discontinued HD after completely recovering from COVID-19. All patients received an antiviral agent, whereas 28.6% of the patients received intensive care, including mechanical ventilation and continuous renal replacement therapy for a median duration of more than one week with one patient receiving extracorporeal membrane oxygenation. Among the included patients, 64.3% improved and were discharge from the hospital after a median length of stay of 22.0 days. However, some patients on HD, particularly those with severe pneumonia upon hospitalization, died from COVID-19 despite prompt critical care. Overall, COVID-19 outcomes among patients on HD were relatively acceptable. Favorable outcomes could be attributed to prompt diagnosis, hospitalization, and early treatment within 3.5 days after symptom onset.

Although four of the patients included had a definite history of contact with a patient suffering from COVID-19, transmission routes for the other patients remained uncertain. As patients receiving maintenance HD shared densely populated spaces during dialysis, all patients on HD and medical staff in each initial dialysis center, where COVID-19 had been identified, were tested for SARS-CoV-2. Accordingly, three medical staff from two private dialysis centers were diagnosed with COVID-19, perhaps given their close proximity with patients during dialysis procedures. Fortunately, no infection transmission occurred between patients. The Korean Society of Nephrology had released clinical practice guidelines for preventing COVID-19 transmission within hemodialysis facilities in 2020 [17]. The availability of a systematic management system for patients on dialysis after the COVID-19 outbreak may have contributed to the prevention of inter-patient transmission of infections. After patients on HD with confirmed COVID-19 had been transferred to tertiary university hospitals, remaining patients undergoing HD in each initial dialysis center, where COVID-19 had been identified, received cohort-isolated dialysis. Such prompt and extensive examination and cohort isolation of contact persons may have reduced the additional spread of COVID-19 among patients on HD.

Contrary to previous studies on the general population wherein more men than women had been infected [5,6], we observed a similar number of cases for both sexes. Studies showed that women generally have reduced susceptibility to viral infections given the protection afforded by the X-chromosome and sex hormones, which play a crucial role in innate and adaptive immunity [18]. However, considering the changes in sex hormones among female patients with end-stage renal disease [19], some distinct differences in demographic findings may be present compared with the general population. Further studies, including a larger number of patients with COVID-19 undergoing dialysis, are required to obtain comprehensive demographic findings and identify associated mechanisms.

The most common symptoms observed were respiratory symptoms, such as cough, sputum production, and dyspnea. A previous study including five patients on HD with COVID-19 revealed that diarrhea and non-respiratory symptoms were the most common symptoms [11]. Another case report similarly reported that nausea and vomiting were the first symptoms exhibited by patients on HD with COVID-19 [12]. In the present study, only two patients developed gastrointestinal symptoms, which are not uncommon for patients with COVID-19 [20]. Our patients were diagnosed and admitted to the hospital 2.6 and 3.5 days after symptom onset, respectively. Patients on HD had a shorter mean symptom duration before admission compared to the general population, which was reported to be seven days [6], perhaps due to their frequent hospital visitations and medical staff meetings.

All patients received isolated HD in a negative pressure room. During HD, patients wore surgical masks, while medical staff wore N95 facial masks, eye shields, gowns, caps, and gloves. The majority of the patients received both antiviral and antibacterial agents. The patients had highly elevated inflammatory markers, including C-reactive protein and ferritin, suggesting virally driven hyperinflammation. A study suggested the clinical significance of concentrations of 25-hydroxyvitamin to control inflammatory responses to COVID-19 [21]. Lymphopenia, which was identified as a predictor of COVID-19 severity [22] during the early period, tended to improve over time, whereas procalcitonin, a protein marker suggesting a bacterial infection [23], remained elevated in some of our patients. Although it remains unclear whether elevated procalcitonin is due to a bacterial coinfection or severe COVID-19, it may guide treatment and serve as a predictor of outcome. Considering a recent meta-analysis on COVID-19 reporting that procalcitonin >0.5 µg/L is associated with an almost five times higher risk of severe infection compared to lower levels [24], procalcitonin levels should not be overlooked, even among patients with COVID-19. As such, patients with COVID-19 who exhibit elevated procalcitonin need to be treated with proper antibacterial agents.

Despite critical care, including mechanical ventilation, continuous renal replacement therapy, and extracorporeal membrane oxygenation, two of our patients (both women aged 56 and 64, with no other pre-existing diseases other than hypertension) died due to hypoxemia, unsolved lung lesions, and persistent severe respiratory between 18 February and 14 April 2020. As both patients had been diagnosed and hospitalized six to seven days after symptom onset and had severe pneumonia upon hospitalization, both deaths may have been linked to the relatively delayed diagnosis and hospitalization. This suggests that early diagnosis and prompt medical action are crucial for better outcomes, especially among patients on dialysis with COVID-19. More than half of the patients discharged from the hospital had a stable general condition. Patients on HD took longer to be released from quarantine and discharged (median of 21.0 days) compared to the critically ill patients with COVID-19 not undergoing dialysis (median hospital stay of 17 days) [25]. Notably, one patient in their 80s had recovered from COVID-19 and was discharged from the hospital. Several reasons can explain the successful recovery from COVID-19 among patients on HD despite the high-risk conditions, including pre-existing comorbidities and older age. First, diagnosis, hospitalization and treatment had been conducted within three days, on average, after symptom onset. Evidently, optimal supportive care to maintain proper oxygenation and vital signs could contribute to favorable outcomes. Second, whereas Chinese studies provided antiviral agents to only 35.8–89.9% of their patients [5,6,10], all patients included herein received antiviral agents. We speculate that early administration of antiviral agents among patients on dialysis may be associated with improved outcomes. However, further randomized controlled studies would be required to clarify the effect of early antiviral administration in COVID-19.

This descriptive study has several limitations worth noting. First, we were unable to determine the differences in clinical characteristics and laboratory findings between survivors and non-survivors due to the limited number of patients. Second, other viral, bacterial, or fungal pathogens were not actively identified. Third, nationwide data regarding patients on HD with confirmed COVID-19 are required to precisely determine the mortality and recovery rates within the dialysis population. Fourth, there are no data on the presence of antibodies (IgG, IgM antibodies for SARS-CoV-2) over time confirmed by serological tests, which may have yielded more useful information for the evaluation of a normal immune response and a possible response to the vaccine in patients on HD. Nevertheless, the current study describes in detail the clinical characteristics, laboratory and radiologic findings, courses, and outcomes of patients on HD with COVID-19.

Although some of the patients on HD, especially those already having severe pneumonia upon hospitalization, died due to COVID-19 despite prompt critical care, overall outcomes of COVID-19 among patients on HD remained favorable than expected, perhaps owing to early diagnosis, hospitalization, and antiviral management. Further studies including a larger number of patients on dialysis with COVID-19 are needed to fully understand the clinical features and outcomes of patients on HD.

## Figures and Tables

**Table 1:** Clinical characteristics.

Variable	Patients (*n* = 14)
Age	
Mean age (range), years	63.5 ± 14.5 (40.0–88.0)
Distribution, *n* (%)	
<50 years	2 (14.3)
50–64 years	8 (57.1)
65–79 years	1 (7.1)
≥80 years	3 (21.4)
Sex, *n* (%)	
Male/Female	6 (42.9)/8 (57.1)
Mean duration of dialysis (range), years	4.7 ± 5.3 (0–21.0)
Cause of ESRD, *n* (%)	
Diabetes mellitus	7 (50.0)
Hypertension	2 (14.3)
Others	5 (35.7)
Body mass index, kg/m^2^	23.6 ± 4.3
Comorbidities, *n* (%)	
Cardiovascular disease	2 (14.2)
Cerebrovascular disease	3 (21.4)
Chronic obstructive pulmonary disease	0
Diabetes mellitus	7 (50.0)
Hypertension	11 (78.6)
Cancer	2 (14.3)
Charlson Comorbidity Index	5.6 ± 1.8
History of contacts with patient with COVID-19, *n* (%)	4 (28.6)
Symptoms	
Cough	7 (50.0)
Sputum	3 (21.4)
Dyspnea	5 (35.7)
Sore throat	1 (7.1)
Rhinorrhea	2 (14.3)
Myalgia	2 (14.3)
Fatigue	4 (28.6)
Nausea, vomiting, or diarrhea	2 (14.3)
Mean duration from symptom onset to diagnosis, days	2.6 ± 2.3
Mean duration from symptom onset to admission, days	3.5 ± 2.8

Abbreviations: ESRD, end-stage renal disease; COVID-19, coronavirus disease 2019; IQR, interquartile range. Data are presented as mean ± standard deviation with/without range, median (IQR), or *n* (%).

**Table 2:** Vital signs, laboratory data, and imaging findings on admission.

Parameter	Patients (*n* = 14)
Vital signs	
Systolic blood pressure, mmHg	136.5 ± 24.7
Diastolic blood pressure, mmHg	76.8 ± 15.6
Heart rate >100 beats per min, *n* (%)	2 (14.3)
Respiratory rate ≥20 breaths per min, *n* (%)	5 (35.7)
Body temperature ≥ 38 °C, *n* (%)	3 (21.4)
Laboratory results (normal range)	
White blood cell count, ×10^3^/µL (4.0–10.0)	5.8 ± 2.1
Lymphocyte count, ×10^3^/µL (1.0–4.5)	1.1 ± 0.5
Hemoglobin, g/dL (13.0–18.0)	10.5 ± 1.4
Platelet count, ×10^3^/µL (130–400)	162.1 ± 74.0
C-reactive protein, mg/dL (<0.5)	6.1 (3.0–8.8)
Ferritin, ng/mL (*n* = 9; 30–400)	472.0 (292.4–1693.2)
Procalcitonin, ng/mL (*n* = 9; <0.05)	0.6 (0.3–1.0)
Albumin, g/dL (3.5–5.2)	3.3 ± 0.7
Alanine aminotransferase, U/L (<41)	14.5 (11.5–18.0)
Aspartate aminotransferase, U/L (<40)	19.0 (16.0–33.8)
Lactate dehydrogenase, U/L (135–250)	264.5 (196.0–401.0)
Creatine phosphokinase, U/L (*n* = 10; 26–192)	210.4 ± 188.8
Lactic acid, mmol/L (*n* = 8; 0.5–1.6)	1.3 (1.1–2.2)
Prothrombin time, sec (*n* = 12; 10–14)	13.1 (13.0–13.9)
Activated partial thromboplastin time, s (*n* = 12; 20–35)	37.8 (36.3–40.9)
D-dimer, µg/mL (*n* = 7; <0.5)	1.7 (1.0–3.8)
Chest radiography findings, *n* (%)	
Clear	4 (28.6)
Unilateral infiltration	3 (21.4)
Bilateral infiltration	7 (50.0)
Pleural effusion	2 (14.3)

Abbreviations: COVID-19, coronavirus disease 2019; IQR, interquartile range. Data are presented as means ± standard deviation, median (IQR), or *n* (%).

**Table 3:** Treatment.

	Patients (*n* = 14)
Treatment, *n* (%)	
Low-flow oxygen therapy	4 (28.6)
High-flow oxygen therapy	2 (14.3)
Invasive mechanical ventilation	4 (28.6)
ECMO	1 (7.1)
CRRT	4 (28.6)
Lopinavir and ritonavir	14 (100.0)
Hydroxychloroquine	7 (50.0)
Others	
Antibiotics treatment	13 (92.9)
Antifungal treatment	2 (14.3)
Glucocorticoids	3 (21.4)
Intravenous immunoglobulin therapy	1 (7.1)
Vasopressors	4 (28.6)
Events during hemodialysis, *n* (%)	
Hypotension	3 (21.4)
Dialysis vascular access occlusion	3 (21.4)
Arrhythmia	1 (7.1)

Abbreviations: ECMO, extracorporeal membrane oxygenation; CRRT, continuous renal replacement therapy. Data are presented as *n* (%).

**Table 4:** Clinical outcomes.

	Patients (*n* = 14)
Outcomes	
Median length of stay (IQR), days	
In hospital	22.0 (14.0–30.0)
In ICU (*n* = 5)	6.0 (5.0–27.0)
Median duration of positive results following COVID-19 test (IQR), days	21.0 (12.0–30.0)
Median duration of mechanical ventilation (IQR), days (*n* = 4)	14.0 (4.0–28.0)
Median duration of ECMO, days (*n* = 1)	3
Median duration of CRRT (IQR), days (*n* = 4)	8.5 (3.0–25.0)
Died in the hospital, *n* (%)	2 (14.3)
Discharged from the hospital, *n* (%)	9 (64.3)
Remained in the hospital, *n* (%)	3 (21.4)

Abbreviations: IQR, interquartile range; ICU, intensive care unit; COVID-19, coronavirus disease 2019; ECMO, extracorporeal membrane oxygenation; CRRT, continuous renal replacement therapy. Data are presented as median (IQR) or *n* (%).
